# Crystal design using multipolar electrostatic interactions: A concept study for organic electronics

**DOI:** 10.3762/bjoc.9.272

**Published:** 2013-11-05

**Authors:** Peer Kirsch, Qiong Tong, Harald Untenecker

**Affiliations:** 1 Merck KGaA, Liquid Crystal R&D Chemistry, Frankfurter Str. 250, D-64392 Darmstadt, Germany, Tel: (+49)6151-72-41118, Fax: (+49)6151-72-2593

**Keywords:** aromatic stacking, charge carrier transport, crystal design, electrostatic control, organic semiconductor, organo-fluorine

## Abstract

Using a simple synthetic protocol, heterohexacene analogues with a quadrupolar distribution of partial charges are readily available. In contrast to most other acenes, these compounds crystallize with a slipped-stack, brickwork-like packing which is mainly controlled by electrostatic interactions. This type of packing offers an advantage for organic semiconductors, because it allows more isotropic charge transport compared to the “herring bone” stacking observed for other acenes.

## Introduction

Within a very few years the first organic semiconductors have found practical application in printed circuits for driving e-paper displays [1–2]. Among the most critical parameters for their application in organic field effect transistors (OFET) are their charge carrier mobility and their solubility in non-toxic organic solvents for processing by printing techniques [3]. High charge carrier mobilities are a particularly critical prerequisite for application in backplanes for OLED displays.

The highest charge carrier mobilities in organic compounds have so far been reported for single crystals of small molecules [4], the “classics” among them being acenes such as pentacene [5], which act as *p-*type semiconductors. However, acenes in the crystalline state tend to form slightly tilted molecular stacks (“herring bone” pattern), which is resulting in a strong directional anisotropy for the charge carrier mobility along the stacking direction. Charge carrier transport in organic materials, or electron transport in general, occurs in most cases by a hopping mechanism which is described by the Marcus theory [6].

[1]
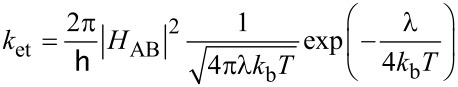


According to Marcus’ formula (1), the rate of electron transfer (*k*_et_) between equal molecules is controlled by the electronic overlap (transfer integral *H*) between the donating and the receiving orbital, which is very sensitive to intermolecular distance and relative orientation [7]. The second parameter determining the charge transfer rate is the reorganization energy (λ), required to accommodate the newly charged or discharged molecule within the crystal lattice. Charge carrier mobility can be optimized by minimizing the reorganization energy and by maximizing the transfer integral. Low reorganization energies are generally realized very well by the higher acenes, due to their wide charge delocalization und their small differences in geometry between the neutral species and the charged radical.

**Figure 1:**
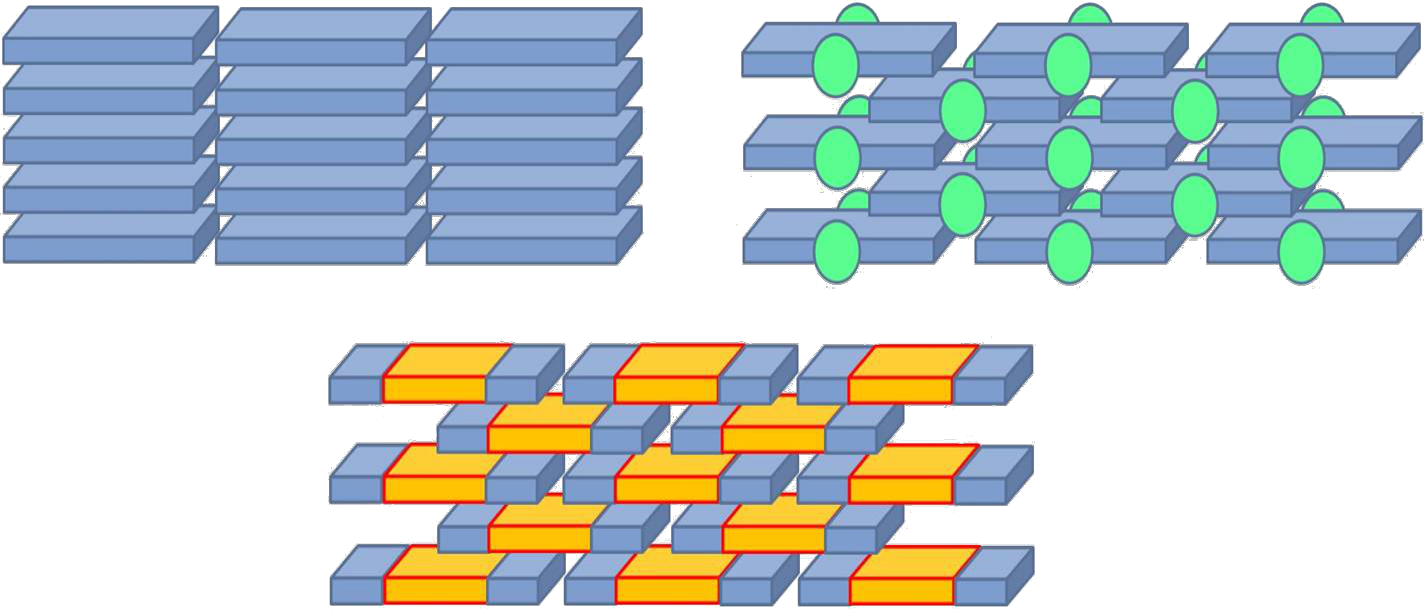
Schematic view of the different types of molecular arrangements in acene-based molecular semiconductors: "herringbone" stacking (upper left) vs sterically induced slipped stack, "brick-like" TIPS-pentacenes (upper right, the green spheres denote sterically demanding lateral groups) vs electrostatically induced slipped stack concept (bottom, the different colors denote opposite partial charges on the electrostatic potential surface).

However, the stacked crystal structure found in acenes in general results in an extremely one-dimensional directionality of charge transport within the single stacks with its inherent vulnerability towards even minor structural defects. Thus, the experimentally observed charge carrier mobilities, e.g., in pentacene based OFETs, remain far below the theoretically predicted limit of several tens of cm^2^·V^−1^s^−1^ [8]. In order to overcome this obstacle, it would be ideal to have acene-like organic semiconductors which pack in the crystal not in one-dimensional stacks but in a brickwork-like pattern with two-dimensional overlap. This has been very efficiently realized by Anthony et al. for pentacene derivatives by attaching sterically demanding trialkylsilylacetylene moieties to the central ring, forcing the crystal into a brick-like arrangement (Figure 1) [9]. However, the price to pay for this optimized morphology is the presence of bulky silyl groups which do not electronically contribute to the charge transport.

For this reason we set out to execute an alternative concept to achieve a brick-like, slipped stacking in planar, acene-based organic semiconductors. In contrast to Anthony’s approach of steric interference with the formation of one-dimensional stacks, our approach is based on “sculpting” the electrostatic potential surface of the semiconductor molecule in a way that cofacial stacking leads to electrostatic repulsion which can be converted into attraction by sliding the π-systems against each other by about half a molecular length. The advantage of this concept is not only a brick-like 2D structure, but also supposedly a tighter packing with smaller interplanar distances due to the strong electrostatic interactions. A similar effect has been demonstrated by Watson et al. [10] with bipolar, partially fluorinated aromatic compounds, which form closely packed 1D stacks through self-complementary structure of their electrostatic potential surface.

**Figure 2:**
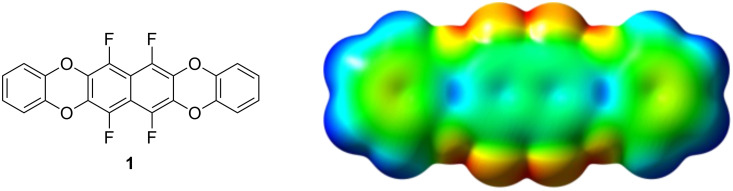
Target compound **1** and its calculated electrostatic potential surface. The colors denote a range of partial charges between −0.033 *e* (red) to +0.023 *e* (blue) [15].

Chemically, the electrostatic potential surface can be modelled very efficiently with partially fluorinated arenes, without expanding the geometry too much. Arene–perfluoroarene interactions are well known to stabilize molecular crystals [11–13] through multipolar electrostatic interactions, and there are a few examples for their use in organic electronics [10,14]. Another point to consider are the HOMO and LUMO energy levels, which have to be consistent with the work function of electrode materials used in OFETs – typically gold with a work function of around −5.4 eV. Taking this into account, the semifluorinated tetraoxatetrahydrohexacene **1** was selected as synthetic target for our concept study, with a predicted [15] HOMO energy of −5.64 eV and LUMO of −1.29 eV (Figure 2).

## Results and Discussion

### Synthesis and structural characterization

**Scheme 1:**
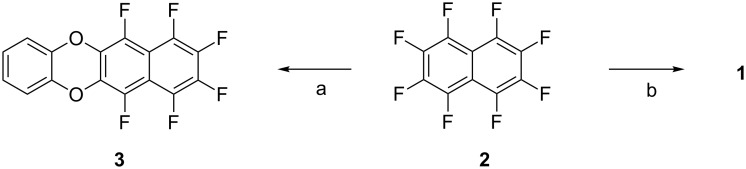
Syntheses of the substitution products **1** and **3**: a) Catechol, K_2_CO_3_, THF; 60 °C, 4 h (12%). b) Catechol, K_2_CO_3_, DMEU; 90 °C, 18 h (44%).

The synthesis of model compound **1** is extremely simple: octafluoronaphthalene (**2**) [16–18] is reacted with catechol in the presence of potassium carbonate (Scheme 1). Under relatively mild conditions (THF, 60 °C, 4 h) only the *mono-*substitution product **3** is formed, whereas at a higher temperature (DMEU, 90 °C, 18 h) the target compound **1** is furnished in moderate yield. The solubility of **1** in all common organic solvents was found to be extremely poor and impeded the purification as well as the analytical characterization. Attempts to purify the material by sublimation resulted only in its decomposition.

It is well known, that octafluoronaphthalene (**2**) reacts quite selectively towards nucleophilic attack [16–18]. Under mild reaction conditions normally the fluorine atom at the 2-position is replaced first, followed by the fluorine atom at the 7-position. If the temperature is raised, the fluorine atoms at the 3- and the 6-positions are substituted next. This selectivity can be explained by an analysis of the partial charge distribution in **2** [15]. The carbon atoms at the 2-, 3-, 6- and 7-positions carry positive partial charges of +0.508 *e*, whereas the *peri-*carbons are much less positive with only +0.276 *e*. This renders the *peri-*position less susceptible to the charge-controlled attack by hard nucleophiles.

**Figure 3:**
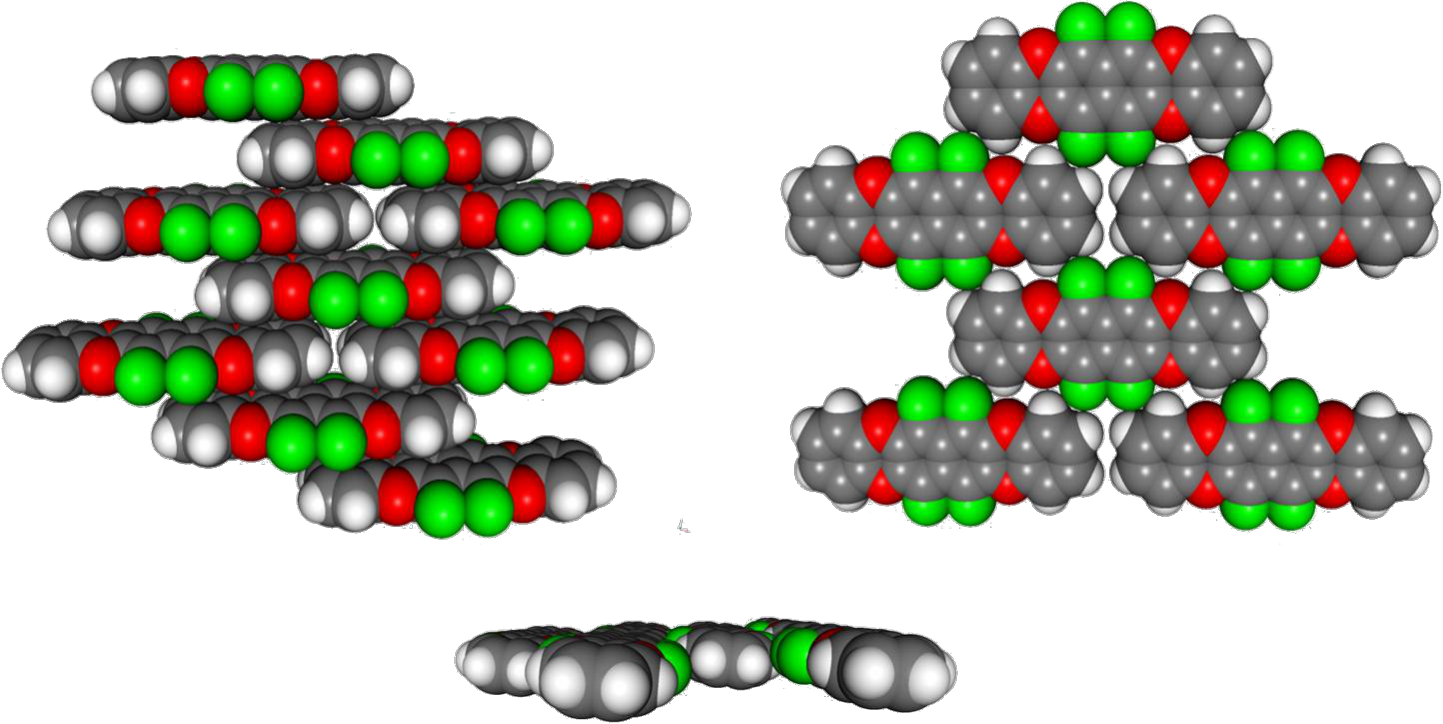
The crystal structure of **1** is characterized by brick wall-like stacks (left), which are arranged in sheets (right). Within the sheets the molecules are nearly parallel, slightly tilted along their long axis by ca. 5.2° (bottom).

In spite of the extremely poor solubility of **1** in all usual organic solvents, small crystals suitable for X-ray structure analysis were obtained by very slow room temperature crystallization from THF. The quality of the crystals was borderline poor, but it was possible to gather data for a sufficiently accurate picture of the packing pattern of the compound (Figure 3).

**Figure 4:**
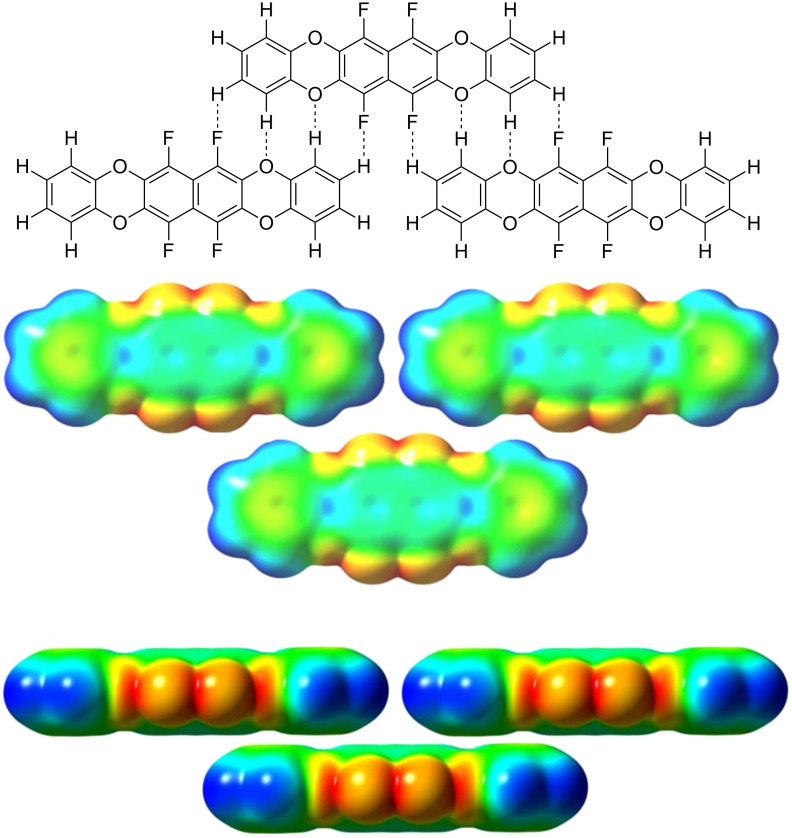
The electrostatic factors determining the packing of **1**. The laterally interlinked sheets are stabilized by dipolar H···O and H···F bridges. The inter-plane arrangement is dominated by the complementary quadrupolar, partial charge distribution on the surface of **1**.

The packing of **1** shows two dominant features: one is the slipped-stack motif of the arenes with an average interplanar distance of 363 pm. The slippage is not exactly half a period as intended, but slightly more. However, it clearly reflects the quadrupolar pattern of charge distribution on the surface of **1**. The other feature are laterally interlocking sheets with close contacts between electropositive hydrogen and electronegative fluorine (H···F 260.6 pm) and oxygen (H···O 279.1 pm). In contrast to most other acenes, such as hexacene [19], the stacks are only very slightly tilted around their long axis (only ca. 5.2° relative to the sheet plane) (Figure 4).

The reason why **1** does not display the acene-typical herring bone arrangement appears to be the distribution of the electrostatic potentials dominating the packing completely. However, a drawback of the electrostatically controlled crystal packing is the very poor solubility of **1**.

### Theoretical study on electronic properties

**Figure 5:**
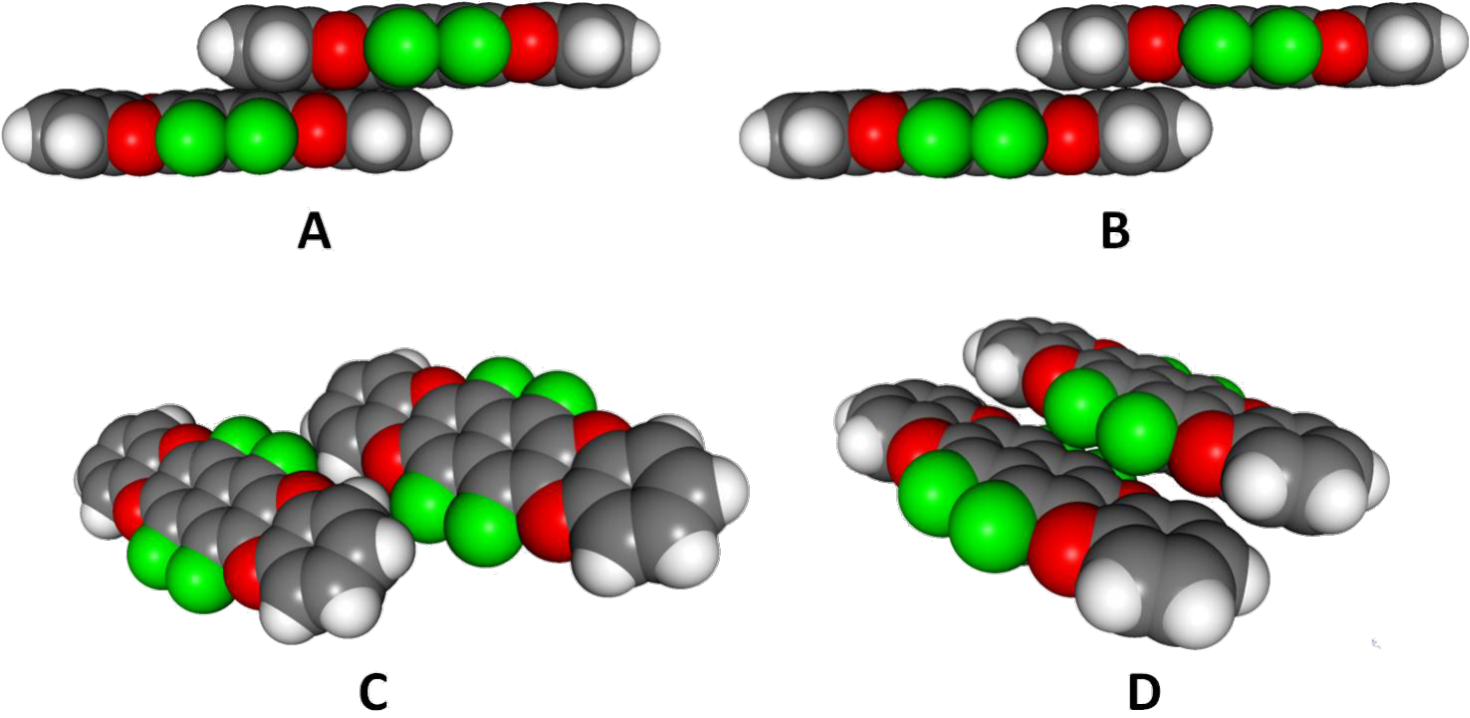
The four closest pairs **A**–**D** in the crystal structure of **1**. The corresponding transfer integrals for hole (*H*_+_) and electron tranport (*H*_−_) are as follows: **A**: *H*_+_ = 36 meV, *H*_−_ = 33 meV; **B**: *H*_+_ = 0 meV, *H*_−_ = 24 meV; **C**: *H*_+_ = 1 meV, *H*_−_ = 3 meV; **D**: *H*_+_ = 3 meV, *H*_−_ = 20 meV [15].

In order to explore the potential utility of **1** and its analogues as an organic semiconductor, the transfer integrals [20–21] for hole and electron transfer between the four closest pairs of molecules were calculated (Figure 5).

**Figure 6:**
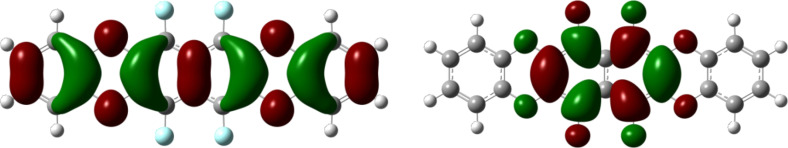
Geometries of HOMO (−5.64 eV, left) and LUMO (−1.29 eV, right) of **1** [15].

The analysis shows that particularly the hole transfer proceeds predominantly through the π-faces (mainly pair **A**), limiting the charge carrier transport to the π-stacked "brick wall". For electron transport the pair **D** allows additional charge transfer between adjoining "walls". This difference between hole and electron transfer can be explained by the geometries of the HOMO and LUMO orbitals (Figure 6) and their different modes of overlap in the arrangements **A**–**D**. Whereas the HOMO is all over the aromatic part of **1**, the LUMO is more contracted to the central naphthalene unit, but extends also to the four fluorine substituents. This is probably the reason for the significant lateral electron mobility between the adjoining "brick walls" in contrast to the hole mobility which is limited to one contact (**A**) with particularly strong π–π overlap.

In conclusion, **1** can be considered predominantly as a highly anisotropic 1D conductor for holes, but as a quite isotropic 3D conductor for electrons.

## Conclusion

The fluorinated tetraoxatetrahydrohexacene derivative **1** was synthesized in an extremely simple, one-step procedure from octafluoronaphthalene and catechol. Due to the quadrupolar distribution of partial charges, the compound does not crystallize with the herring bone packing typical for other acenes. It forms a brickwork-like assembly of the aromatic units, which is laterally integrated into a structure of interlocked sheets, stabilized by spatial and electrostatic H∙∙∙F and H∙∙∙O contacts. The calculation of transfer integrals based on the crystal structure indicates that in spite of the brickwall-structure, due to a slight asymmetry of the “layering”, the hole conductivity is rather anisotropic (1D), whereas the electron conductivity is three-dimensional (3D). Although the control of the crystal packing by “sculpting” the electrostatic potential surface is demonstrated impressively, also the drawback of the concept becomes visible: the same intermolecular electrostatic forces which shape and stabilize the crystal packing cause extremely poor solubility and processability.

## Experimental

**General remarks:** Reagents and solvents were obtained commercially and used as supplied. ^1^H and ^19^F NMR spectra were collected using a Bruker Avance 400 spectrometer. ^1^H NMR chemical shifts were referenced to the solvent signal. GC–MS experiments were performed on an Agilent 7890A system equipped with a 7000A Triple Quad detector. Melting points were determined by differential scanning calorimetry (DSC) performed on a Universal V4.5A (TA Instruments) at a heating/cooling rate of 20 K∙min^−1^. The temperature was calibrated with indium. The measurements were performed under a nitrogen atmosphere. X-ray diffraction measurements were carried out with a SuperNova (Agilent) diffractometer.

**Synthesis of 6,7,8,9,10,11-hexafluorobenzo[*b*]oxanthrene (3):** A solution of octafluoronaphthalene (**2**, 340 mg, 1.2 mmol) in THF (10 mL) was added dropwise to the suspension of catechol (0.26 g, 2.4 mmol) and potassium carbonate (410 mg, 2.9 mmol) in THF (30 mL) at 60 °C. After complete addition, the reaction mixture was stirred at 60 °C for another 4 hours. The reaction mixture was allowed to cool down to room temperature and filtrated. The solid residue was washed repeatedly with THF, and the filtrate was evaporated to dryness in vacuo. The remaining crude product was purified by recrystallization from isopropanol/THF (2:3) solvent mixture. After drying in vacuo for 18 hours the product was obtained as colourless crystalline solid. Yield: 46 mg (12%) of colorless crystals, mp 258 °C. ^1^H NMR (400.1 MHz, THF-*d*_8_) δ 7.15–7.06 (m, 4H); ^19^F NMR (376.4 MHz, THF-*d*_8_) δ −148.23 to −148.39 (m, ^4^*J*_FF_ = 61 Hz, 2F), −148.80 to −149.01 (m, ^4^*J*_FF_ = 61 Hz, ^3^*J*_FF_ = 15 Hz, 2F), −159.61 to −159.65 (m, ^3^*J*_FF_ = 15 Hz, 2F); MS (HPLC–APLI) *m*/*z* (%): 342 [M]^+^ (100); HRMS (ASAP–MS, 100–500 °C, C_22_H_8_O_4_F_4_): calcd 412.0358798; found, 412.03517; Single crystals for the X-ray structure analysis were obtained by slow crystallization from THF. Crystal structure data for **1** (C_22_H_8_F_4_O_4_): crystal size 0.0909 × 0.045 × 0.0297 mm, triclinic, *P−*1, *a* = 6.0570(9) Å, *b* = 7.6382(12) Å, *c* = 8.829(3) Å, α = 94.98(2)°, β = 97.72(2)°, γ = 101.372(13)°, *V* = 394.11(16) Å^3^, *Z* = 1, ρ_calcd_ = 1.737 g·cm^−1^, *R*(F) = 5.94% for 754 observed independent reflections (5.09° ≤ 2θ ≤ 50.36°). Crystallographic data for the structure reported in this paper have been deposited at the Cambridge Crystallographic Data Centre as supplementary publication No. CCDC-947860. Copies of the data can be obtained free of charge on application to CCDC, 12 Union Road, Cambridge CB2 1EZ, UK (fax: (+44) 1223-336-033; e-mail: deposit@ccdc.cam.ac.uk; http://www.ccdc.cam.ac.uk/).

**Synthesis of 6,7,14,15-tetrafluorooxanthreno[2,3-*b*]oxanthrene (1):** A solution of octafluoronaphthalene (**2**, 340 mg, 1.2 mmol) in 1,3-dimethylimidazolidinone (DMEU, 5 mL) was added dropwise to the suspension of catechol (0.26 g, 2.4 mmol) and potassium carbonate (630 mg, 4.3 mmol) in DMEU (15 mL) at 90 °C. The reaction mixture was stirred at 90 °C for 18 hours. After completion of the reaction, the reaction mixture was cooled down to room temperature and poured into a mixture of ice and water. The precipitated crude product was filtrated and purified by recrystallization from benzonitrile. After drying in vacuo for 18 hours the product was obtained as white crystalline solid. Yield: 190 mg (44%) of colorless crystals, mp 302 °C. The solubility in THF-*d*_8_ and other suitable solvents was not sufficient for obtaining meaningful solution NMR spectra. MS (HPLC-APLI) *m*/*z* (%): 412 [M]^+^ (100).
